# Antimicrobial activity of probiotic irrigant with and without *mentha piperata* essential oil against *E. faecalis*: An *In vitro* comparative study

**DOI:** 10.6026/973206300220186

**Published:** 2026-01-31

**Authors:** Anuja Ajaykumar Dhumal, Damini Patil, Akshay Arun Khase, Priyanka Dhananjay Kombade, Bhagyashri Narayan Magar, Yogesh Balaji Chanchalwad

**Affiliations:** 1 Department of Conservative Dentistry and Endodontics, Bharati Vidyapeeth Deemed to be University Dental College and Hospital, Sangli, Maharashtra, India; 2 Department of Conservative Dentistry and Endodontics, Y.M.T. Dental College, Kharghar, Navi Mumbai, Maharashtra, India; 3 Department of Conservative Dentistry and Endodontics, MIDSR, Latur, Maharashtra, India; 4 Department of Conservative Dentistry and Endodontics, SB Patil Dental College and Hospital, Bidar, Karnataka, India; 5 Department of Conservative Dentistry and Endodontics, School of Dental Sciences Krishna Vishwa Vidyapeeth, Malkapur, Karad, Maharashtra, India

**Keywords:** Probiotic irrigant, *mentha piperata* essential oil, *E. faecalis*, *Lactobacillus rhamnosus*

## Abstract

Persistent *Enterococcus faecalis* infections in root canals following endodontic therapy pose significant treatment challenges due to its antimicrobial resistance and biofilm formation. Therefore, it is of interest to evaluate the antimicrobial activity of probiotic irrigant (*Lactobacillus rhamnosus*) with and without *mentha piperata* essential oil against *E. faecalis* by measuring zones of inhibition on Mueller Hinton agar and blood agar plates at 24- and 48-hour intervals. *E. faecalis* was cultured on agar plates, and three irrigant solutions were tested: 5.25% sodium hypochlorite (Group 1), probiotic irrigant alone (Group 2) and probiotic irrigant with mentha piperata essential oil (Group 3). Data shows that probiotic irrigant combined with *mentha piperata* essential oil (Group 3) exhibited significantly larger zones of inhibition compared to sodium hypochlorite alone (Group 1) on both media, while probiotic irrigant without essential oil (Group 2) showed comparable inhibition to sodium hypochlorite. Probiotic irrigant combined with Mentha piperita essential oil demonstrates superior antimicrobial efficacy against *E. faecalis*, with recommendations for future *In vivo* studies to evaluate effectiveness under complex oral conditions.

## Background:

Infections inside the root canals, outside the root canals, reactions to foreign bodies, cysts containing cholesterol crystals, and extra radicular infections are the causes of chronic periradicular infection after root canal therapy [1]. The survival of microbes in the apical region of the root is often regarded to be the major cause of failure. The anaerobic, facultative bacterium Enterococcus faecalis is often present in the apical one third of root canal failure cases and is very resistant to traditional chemo-mechanical preparation [2]. Endodontic therapy aims to eliminate pulpal wastes, germs, and byproducts from diseased root canals and disinfected root canals as a whole [3]. Over the last several decades, 5.25% sodium hypochlorite (NaOCl) has been widely used as the primary irrigant for root canal therapy [4]. The dissolving of organic tissues is its primary characteristic. *In vitro* tests have shown that sodium hypochlorite effectively kills bacteria, spores, yeast, and viruses [5]. Sodium hypochlorite mishaps may happen, however, if you happen to push it too much past its apex. In light of this limitation, probiotic irrigants may be an alternate course of therapy to investigate. According to the World Health Organization, "probiotics" are living microbes that, when given to the host in sufficient amounts, have a positive effect on the host's health [6]. Members of the genus Lactobacillus are among the most popular probiotic bacterial strains. Enzymes produced by Lactobacillus species aid in the digestion and metabolism of carbohydrates and proteins. Past *In vitro* research has shown that probiotics may be useful in eliminating *E. faecalis* and in root canal treatment [7]. The current investigation included enhancing the efficiency of a probiotic irrigant by adding *M. piperata* essential oil, which has antibacterial properties. Natural and herbal alternatives to synthetic irrigants have gained popularity in recent decades as a result of concerns about their potential negative effects. Plants provide a wealth of therapeutic and antibacterial compounds in the form of essential oils. For those looking for an alternative to NaOCl, there have been studies that found several plant extracts to be effective against *E. faecalis*. Therefore, it is of interest to investigate the efficiency of a probiotic irrigant against *E. faecalis* at two distinct time intervals, with and without the addition of *mentha piperata* essential oil and ]sodium hypochlorite.

## Materials and Methods:

## Study setting:

This present study was done in the Department of Pharmacology affiliated with the Maharashtra University of Health Sciences (M.U.H.S) and is approved by the ethics committee.

## Methods:

Four groups were selected and tested for its antimicrobial activity. For evaluating zone of inhibition following groups were selected:

Group 1- 5.25 % Sodium Hypochlorite

Group 2- *Lactobacillus rhamnosus* with distilled water

Group 3 - *Lactobacillus rhamnosus* with peppermint (*mentha piperata*) essential oil

Group 4 - Control group (Normal Saline)

## Experimental probiotic irrigant preparation:

The probiotic species chosen, *Lactobacillus rhamnosus*, was supplied by the add firm in lyophilized form. A process of anaerobic incubation was used to activate the bacteria by inoculating *L. Rhamnosus* spores into sterile De Man, Rogosa, Sharpe (MRS) broth (Hi-Media, India) for 48 hours at 30°C. To create the two probiotic irrigants that were tested, 5 milliliters of *L. Rhamnosus* active cells were inoculated into 10 milliliters of sterile distilled water, and 5 milliliters of active cells were inoculated into 10 milliliters of peppermint oil at a concentration of 0.55% (v/v). The usual positive control was a solution of NaOCl (5.25%), whereas the negative control was sterile distilled water [8].

## Antimicrobial study:

**Figure 1:**
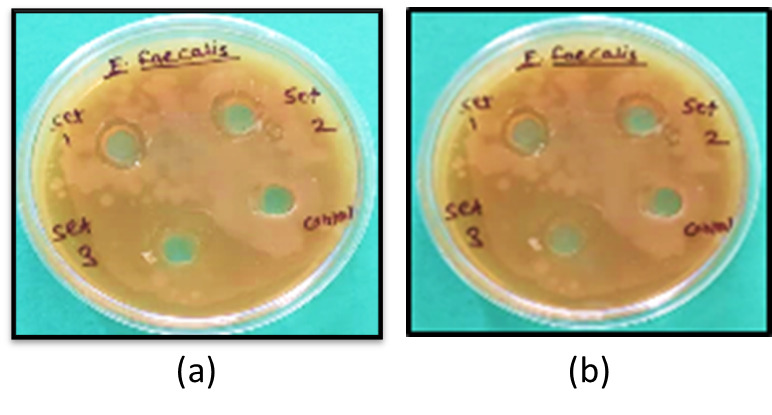
Zone of Inhibition on muller and hinton agar (a) After 24 hours, (b) After 48 hours

**Figure 2:**
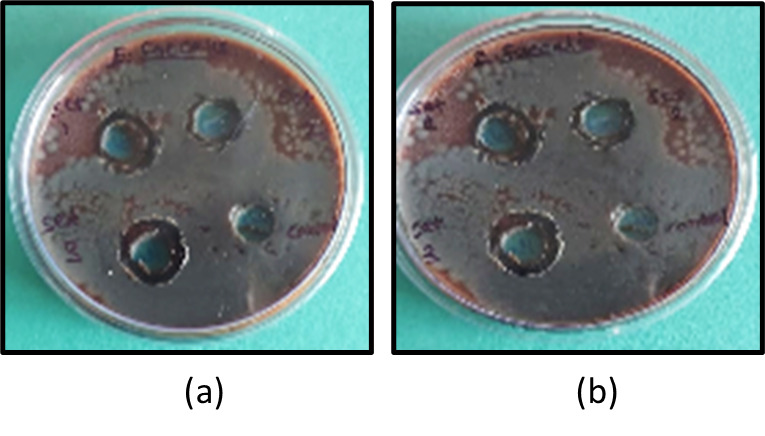
Zone of Inhibition on blood agar (a) After 24 hours, (b) After 48 hours

The pathogenic bacteria, *E. faecalis*, were recently supplied and mixed well in 9 ml of MRS broth using a vortex before being adjusted to a 1 McFarland standard. 100 mm diameter Muller and Hinton Agar plates, blood agar plates, and 500 microliters of *E. faecalis* were disseminated using sterile L-loop. To promote the development of bacterial grass, the samples were cultured for 24 hours. Each test group's prepared irrigant solution was added to a well with a diameter of 0.7 cm using a micropipette, and the mixture was left undisturbed for 5 minutes to let the compounds to diffuse. For each group, the exam was administered three times. At both the 24-and 48-hour intervals, the zones of inhibition (ZOIs) were measured in millimeters using a digital micrometer [8] (Figure 1 & Figure 2).

Group1: Naocl standard

Group 2: L. Rahmnosus with sterile distilled water

Group 3: L. Rahmnosus with 0.55% (v/v) peppermint oil

Group 4: Control - Sterile distilled water

## Results:

The results of the intergroup comparison show that after 24 hrs the inhibition zones of groups 1 and 2 are identical. Group 3 (Probiotic irrigant with menthe piperata essential oil) had a much larger zone of inhibition than Group 1 (Sodium hypochlorite group). The antibacterial activity of the probiotic irrigant has been greatly enhanced by the addition of *mentha piperata* oil. When compared to the other groups, the control group stands out (Table 1, Table 2). After 48 hours, when groups 1, 2, and 3 were compared, there was no significant difference in zone of inhibition. However, when control was compared to the other groups, there was a very significant difference (Table 3, Table 4).

Group 2 (Probiotic irrigant) and Group 1 (Sodium hypochlorite) did not vary significantly in their zones of inhibition when tested on blood agar. There was a statistically significant difference between Group 2 (the probiotic irrigant) and Group 3 (the probiotic irrigant plus *mentha piperata* essential oil). The inhibition zones of groups 1 and 3 are significantly different from one another. The zone of inhibition for the probiotic irrigant containing *mentha piperata* essential oil is much greater than that of the sodium hypochlorite group (Table 5, Table 6). After 48 hours on Blood agar, comparing the zones of inhibition among the three groups shows no statistically significant difference; nevertheless, there is a very significant difference between the control group and every other group (Table 7, Table 8).

## Discussion:

Root canal infections are caused by a small number of species that are not often present in the oral cavity's typical flora. In endodontic illness, the bacterial strain that is regularly detected after therapy, especially in the apical one third of the root, is Enterococcus faecalis. It creates a biofilm, which is a population of microbes adhered to a surface. It is therefore protected from both host defenses and systemic therapies, and it is resistant to both local and systemic treatments [9, 10]. Due of its antibacterial and tissue-dissolving properties, sodium hypochlorite has been the local irrigation solution of choice in endodontics for decades. Its inability to distinguish between necrotic and vital tissues makes it a potential hypochlorite accident trigger; hence measures must be made to stop it from extruding into the periapical area. To sidestep these pitfalls, inexperienced dentists may dilute the drug, reduce its effectiveness, or simply use less volume than prescribed while treating root canals. In light of these limitations, various irrigation systems have emerged. Despite its promise, essential oil-based irrigants and medicaments have received relatively little attention from researchers, despite many studies suggesting they have the ability to reduce the most significant intracanal pathogen, Enterococcus faecalis, and aid in the eradication of intracanal biofilms. Research on the biocompatibility and advantages of endodontic materials based on essential oils is required, according to Marinkovic *et al.* [11]. Probiotics are a major strategy for maintaining good health, according to the World Health Organization. The action mechanism of these probiotics was detailed by Geier. These include producing chemicals that hinder bacteriocin formation, acids and peroxides, changing the local pH, competing for resources, triggering the immune response, and creating physical barriers [12]. These probiotic irrigants were used in conjunction with essential oil of *mentha piperata* to enhance their efficacy in the current investigation.

Essential oils from peppermint *(Mentha piperita L.)* plants, particularly the leaves, contain antimicrobial, antiviral, antioxidant, and antispasmodic properties [13]. Oral dysplastic disease progression has been slowed or halted by an aqueous *Mentha piperita* extract. It is preferable if the antibacterial agent does not hinder the development of the added probiotic irrigant organisms (such as *Lactobacilli rhamnosus*) as the essential oil is recommended at the same time as probiotics. The investigation followed the methodology of Hawrelak *et al.* and employed a Minimum Inhibitory Concentration (0.55 V/V %) to avoid interference [14]. As a result, you may use peppermint essential oil with the probiotic irrigant without worrying about their counteracting each other. In an effort to find an alternative to sodium hypochlorite that would be just as successful, if not more so, in treating endodontic infections while causing less irritation to the periapical tissues, this research set out to investigate this possibility. The zone of inhibition in group 3 was much larger than in group 2 when peppermint oil was added. Probiotic irrigants antibacterial action has been greatly enhanced by *mentha piperata* oil. The antibacterial activity is not seen in the control group. One study found that *M. piperita L.* essential oil effectively inhibited the growth of *S. mutans* and *lactobacilli bacteria* [15, 16]. The antimicrobial properties of peppermint oil extend to Gram-positive and Gram-negative microorganisms [17, 18], so that the probiotic irrigants antibacterial action may have been enhanced. When groups 1, 2, and 3 were examined after 48 hours of time interval, there was no significant difference in zone of inhibition, according to the intergroup comparison.

The results on blood agar were likewise comparable to those on Muller Hinton agar. Sodium hypochlorite group 1 and probiotic irrigant group 2 did not vary significantly in their zones of inhibition, according to an intergroup comparison. The inhibition zone in group 3 has been much enhanced by peppermint essential oil. In comparison to the sodium hypochlorite group, the probiotic irrigant containing *mentha piperata* essential oil had a much larger zone of inhibition on blood agar, suggesting that it had a stronger antimicrobial impact. In contrast to chloramine, hypochlorous acid, and NaOCl, which dissolve tissues, the probiotic irrigant does not inhibit enzyme activity. If groups 1, 2, and 3 are compared after 48, there is no significant difference in the zone of inhibition. The results are in line with the findings of the studies conducted by Bohora *et al.* who found that some strains of Lactobacillus inhibited the development of Escherichia coli [1]. Furthermore, at both the 24-hour and 1-week mark, Rai *et al.* [8] showed that the lactobacilli strains utilized could limit the development of *E. faecalis* and Candida albicans. Within the limitations of this study, it can be concluded that *Lactobacillus rhamnosus* probiotic with *mentha piperata* essential oil showed significantly higher zone of inhibition than 5.25 % NaOCl and probiotic irrigant at both time periods. 5.25 % NaOCl and probiotic irrigant showed insignificant difference in zone of inhibition at both time periods. *mentha piperata* essential oil has significantly improved the zone of inhibition of probiotic irrigant.

## Conclusion:

The potential of a probiotic irrigant including essential oil of *mentha piperata* in root canal treatment is shown. The probiotic irrigant group that included *mentha piperata* essential oil showed more effective antibacterial action than the sodium hypochlorite group when tested against *E. faecalis*. Further *In vitro* and *In vivo* research is required to assess its efficacy against endodontic infectious germs. Thus, *mentha piperata* essential oil mixed with probiotic irrigant might replace chemical disinfectants.

## Figures and Tables

**Table 1:** Zone of inhibition (ZOI) on Muller and Hinton agar after 24 hours measured in mm

		**N**	**Mean**	**SD**	**F**	**p**	**Inference**
Zone of inhibition on Muller and Hinton agar after 24 hours.	Group 1	3	13	1	204	0.00001	Highly significant
	Group 2	3	13	1		(<0.01)	
	Group 3	3	16	1			
	Group 4	3	0	*0.00			
	Total	12	10.5	6.5			
One-way ANOVA test;
* indicates significant difference at p≤0.05

**Table 2:** Post Hoc Tukey's HSD test applied to assess the intergroup difference in ZOI on Muller Hinton agar after 24 hours measured in mm

	**Group 2**	**Group 3**	**Group 4**
Group 1	1	*0.01	*0.0001
Group 2		* 0.012	*0.0001
Group 3			*0.0001

**Table 3:** Zone of inhibition (ZOI) on Muller and Hinton agar after 48 hours measured in mm

		**N**	**Mean**	**SD**	**F**	**p**	**Inference**
Zone of inhibition on Muller and Hinton agar after 48 hours.	Group 1	3	14	1	218.18	0.00001	Highly
	Group 2	3	14	1		(<0.01)	significant
	Group 3	3	16	1			
	Group 4	3	0	*0.00			
	Total	12	11	6.73			
One-way ANOVA test;
* indicates significant difference at p≤0.05

**Table 4:** Post Hoc Tukey's HSD test applied to assess the intergroup difference in ZOI on Muller Hinton agar after 48 hours measured in mm

	**Group 2**	**Group 3**	**Group 4**
Group 1	1	0.085	*0.0001
Group 2		0.085	*0.0001
Group 3			*0.0001

**Table 5:** Zone of inhibition (ZOI) on Blood agar after 24 hours measured in mm

		**N**	**Mean**	**SD**	**F**	**p**	**Inference**
Zone of inhibition on blood agar after 24 hours.	Group 1	3	15	1	235.6	0.00001 (<0.01)	Highly significant
	Group 2	3	13	1			
	Group 3	3	17	1			
	Group 4	3	0	*0.00			
	Total	12	11.25	6.98			
One-way ANOVA test;
* indicates significant difference at p≤0.05

**Table 6:** Post Hoc Tukey's HSD test applied to assess the intergroup difference in ZOI on blood agar after 24 hours measured in mm

	**Group 2**	**Group 3**	**Group 4**
Group 1	0.085	*0.012	*0.0001
Group 2		*0.002	*0.0001
Group 3			*0.0001
Post hoc Tukey's test;
*indicates significant difference at p≤0.05

**Table 7:** Zone of inhibition (ZOI) on Blood agar after 48 hours measured in mm

		**N**	**Mean**	**SD**	**F**	**p**	**Inference**
Zone of inhibition on blood agar after 48 hours.	Group 1	3	15	1	235.6	0.00001 (<0.01)	Highly significant
	Group 2	3	13	1			
	Group 3	3	17	1			
	Group 4	3	0	*0.00			
	Total	12	11.25	6.98			
One-way ANOVA test;
* indicates significant difference at p≤0.05

**Table 8:** Post Hoc Tukey's HSD test applied to assess the intergroup comparison in ZOI on blood agar after 48 hours measured in mm

	**Group 2**	**Group 3**	**Group 4**
Group 1	0.085	0.085	0.0001
Group 2		0.085	0.0001
Group 3			0.0001
Post hoc Tukey's test;
* indicates significant difference at p≤0.05
